# A Review of Ladybug Taint in Wine: Origins, Prevention, and Remediation

**DOI:** 10.3390/molecules26144341

**Published:** 2021-07-17

**Authors:** Gary J. Pickering, Andreea Botezatu

**Affiliations:** 1 Departments of Biological Sciences and Psychology, Brock University, St. Catharines, ON L2S 3A1, Canada; 2 Cool Climate Oenology and Viticulture Institute, Brock University, St. Catharines, ON L2S 3A1, Canada; 3 National Wine and Grape Industry Centre, Charles Sturt University, Wagga Wagga, NSW 2678, Australia; 4 Sustainability Research Centre, University of the Sunshine Coast, Sippy Downs, QLD 4556, Australia; 5 Department of Horticultural Sciences, Texas A&M University, College Station, TX 77843-2133, USA; abotezatu@tamu.edu

**Keywords:** ladybird taint, methoxypyrazines, wine quality, wine faults, grape quality

## Abstract

Ladybug taint (also known as ladybird taint) is a relatively recently recognized fault that has been identified in wines from a wide range of terroirs. Alkyl-methoxypyrazines—particularly 2-isopropyl-3-methoxypyrazine—have been determined as the causal compounds, and these are introduced into grape must during processing, when specific species of vineyard-dwelling Coccinellidae are incorporated into the harvested fruit. *Coccinella septempunctata*, and especially the invasive *Harmonia axyridis*, are the beetles implicated, and climate change is facilitating wider dispersal and survivability of *H. axyridis* in viticultural regions worldwide. Affected wines are typically characterized as possessing excessively green, bell pepper-, and peanut-like aroma and flavor. In this paper, we review a range of vineyard practices that seek to reduce Coccinellidae densities, as well as both “standard” and novel wine treatments aimed at reducing alkyl-methoxypyrazine load. We conclude that while prevention of ladybug taint is preferable, there are several winery interventions that can remediate the quality of wine affected by this taint, although they vary in their relative efficacy and specificity.

## 1. What Is Ladybug Taint?

**Figure 1:**
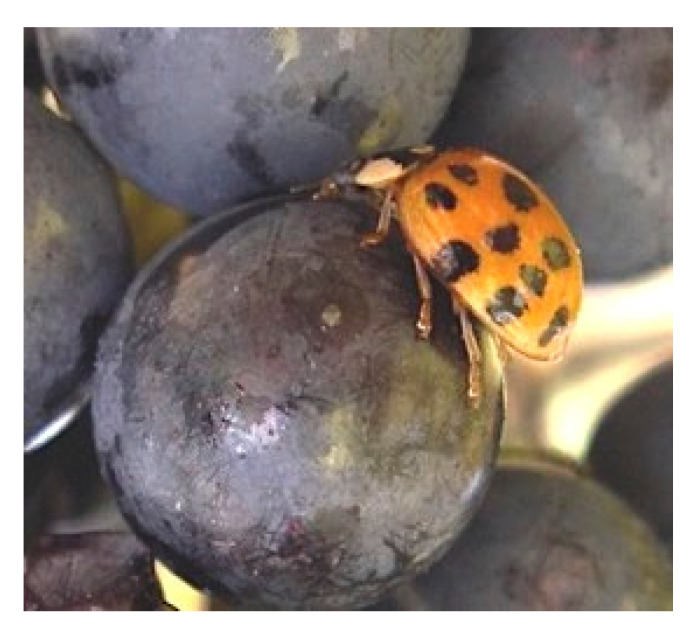
A *Harmonia axyridis* (MALB) beetle on a grape berry prior to harvest.

Ladybug taint (LBT, also known as ladybird taint) was first associated with the presence of large numbers of *Harmonia axyridis* (Pallas) (Coleoptera: Coccinellidae) beetles in vineyards at harvest, and corresponding off-odors in the subsequent wines. *H. axyridis*, more colloquially known as the multicolored Asian ladybeetle (MALB), originates in northeastern Asia, and was first introduced to North America in 1916 as a form of biocontrol against aphids and some small, soft-bodied pests [1,2]. It was introduced to France in 1982 [3], while in Canada it was first reported in southern Québec between 1992 and 1994 [4]. When introduced, the beetle typically extends its range into non-target regions and crops, including grape vineyards, and is now present in many winegrowing countries and regions around the globe. In the vineyard, MALB adults aggregate on grape clusters during autumn, and are often picked with the grapes and transported into the winery [5] (Figure 1). Their presence during crushing and winemaking operations can lead to unpleasant aromas and flavors in the subsequent wine, known collectively as LBT [6,7,8].

Sporadic anecdotal evidence that MALBs have been negatively impacting wine quality has been circulating since the 20th century, but the first widely reported link was noted in 2001 in some North American wine regions, where winemakers described an unpleasant aroma and taste in wines from that vintage similar to “burnt peanut butter” and reminiscent of crushed lady beetles [6]. This observation led to an investigation by Pickering et al. [6], who showed that LBT affects both the aroma and flavor of wines. The authors added MALBs to red and white juice and must, and used descriptive analysis and a trained sensory panel to characterize the wines thus produced. White wines displayed higher intensities of bell pepper, asparagus, and peanut aroma and flavor compared with control wines, while red wines showed higher intensities of peanut, asparagus/bell pepper, and earthy/herbaceous aroma and flavor. At the same time, bitterness (more intense), sourness (more intense), and sweetness (less intense) were also affected in MALB-treated red wines, while in whites the intensity of fruit and floral descriptors was reduced compared with control wines. These effects increased with the number of beetles added to the juice/must [6].

Similar sensory profiles were subsequently reported by Pickering et al. [8,9] and Ross and Weller [10] in other wines produced in the presence of MALBs. The taint appears to be stable over time; affected wines were described similarly at bottling, and again after 10 months of aging [9]. Interestingly, dead beetles are also capable of producing LBT, as demonstrated by the addition of MALBs to red must at various stages postmortem, and subsequent sensory analysis of the wines [7]. At one day postmortem, the beetles negatively influenced the sensory profiles of the wines; however, at three days postmortem and beyond, these effects disappeared. These findings are relevant to tolerance levels for MALB in the winery, as dead beetles can be inadvertently incorporated in with the grapes at harvest—particularly after insecticides having been applied in the vineyard.

### 1.1. Alkyl-Methoxypyrazines Are the Molecules Responsible

**Figure 2:**
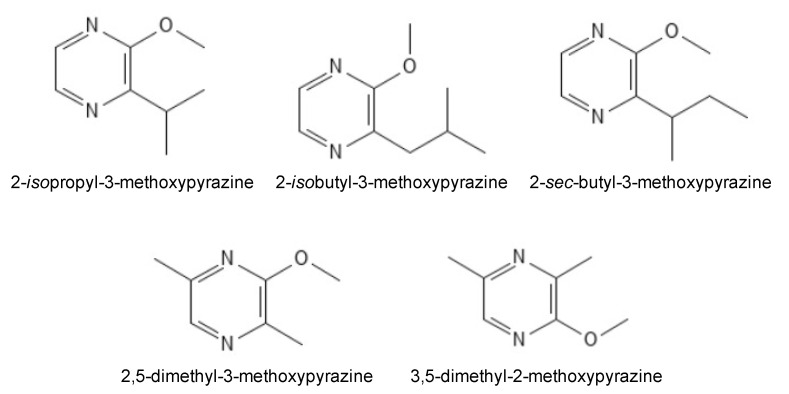
Alkyl-methoxypyrazines identified in Coccinellidae.

Coccinellidae emit a mixture of odor-active compounds that most likely serve several behavioral functions, including defense, aggregation, and mate-attraction. One particular group of compounds—alkyl-methoxypyrazines (MPs)—have been closely scrutinized for their role in LBT (Figure 2). Cai et al. [11] used multidimensional gas chromatography–olfactometry–mass spectrometry to determine the odorants emitted by MALBs, and found that 2-*iso*propyl-3-methoxypyrazine (IPMP) was the most abundant and potent of the pyrazines released. IPMP has been described sensorially as displaying aromas of “peanuts”, “potatoes”, “peas”, and “earthy” [11]. Three more MPs were also identified (2-*iso*butyl-3-methoxypyrazine (IBMP), 2-*sec*-butyl-3-methoxypyrazine (SBMP), and 2,5-dimethyl-3-methoxypyrazine (DMMP)), and their corresponding aromas reported as “peanut, potato, earthy, spicy”, “nutty, potato, peanut”, and “moldy, earthy”, respectively [11], consistent with terms used for describing LBT-affected wines [6,8,9,10]. A more recent study reported a different DMMP isomer—3,5-dimethyl-2-methoxypyrazine—as a component of Coccinellidae hemolymph [12]. IPMP, IBMP, and SBMP have all been detected in the headspace above MALB, with IPMP being the most prevalent [13,14].

Pickering et al. [9] provided the first direct evidence that LBT was caused by MPs. They showed that IPMP levels were higher in white (38 ng/L) and red (30 ng/L) wines fermented with 10 MALB beetles/liter compared with control wines containing no beetles (8 ng/L). Several subsequent studies have confirmed that IPMP concentration in wine increases in the presence of MALBs [7,15,16]. Further implicating the role of IPMP in LBT, Pickering et al. [9] showed that the intensity of earthy/herbaceous descriptors strongly correlates with IPMP concentrations in red wines. Furthermore, wine produced with the addition of MALB and wine spiked with 15 ng/L IPMP display very similar sensory profiles [17].

One study reported no association between IPMP and MALB in wine. Galvan et al. [18] analyzed wines produced from artificially infested Frontenac grapes and from Leon Millot grapes harvested from vineyards with different degrees of “natural” MALB infestation (20% and 50% of the clusters infested with one or more *H. axyridis* adults). They found no differences in concentrations of IPMP between wines with different infestation levels, but they noted that the addition of MALBs did affect the wines’ sensory profiles. The difference between this and previous findings in relation to IPMP may stem from the different analytical techniques employed. SBMP, rather than IPMP, has alternately been suggested as the causal compound for LBT in grape juice [19]; however, the method used to form this conclusion (frequency of detection) is not as robust as other approaches that have used compound concentrations. While Botezatu et al. [20] also reported numerically higher levels of SBMP compared to IPMP in Vidal and Cabernet Sauvignon wines produced with MALB additions, their aroma extract dilution analysis—considered a gold standard for identifying fault compounds in complex matrices—confirmed the dominant role of IPMP. Thus, while other MPs may certainly play a minor part in LBT, the balance of the literature shows that IPMP is the main contributor to the characteristic sensory profile of the taint.

It is important to note that MPs are endogenously produced by certain wine grape varieties; therefore, the presence of MPs in wine is not in and of itself indicative of LBT. In varietals such as Sauvignon Blanc, Cabernet Sauvignon, Cabernet Franc, and Carmenère, these endogenous MPs can confer typicality to their wines at low concentrations, although at elevated levels they negatively impact quality by contributing unbalanced “greenness” and “earthiness” [21,22,23,24,25,26]. In the case of endogenously occurring MPs, IBMP is normally the predominant pyrazine, both in concentration and odor-impact [27,28] It has therefore been proposed that the relative ratio of the different MP species in wine might serve as a “diagnostic” test for Coccinellidae influence. Specifically, the results of Botezatu et al. [28] suggest that an IPMP:IBMP ratio > 1 is indicative of juice/wine contaminated with Coccinellidae beetles.

### 1.2. How Much Is Too Much?

For grape growers and winemakers, it is important to know “how much is too much?”. That is, what densities of MALB in the vineyard should be of concern and necessitate intervention, and what concentrations of MPs are required to elicit LBT in wine?

MPs are extremely odor-active aroma compounds. Most aromatic and volatile components in wine are present and active in the µg/L to mg/L range [29,30], while MP concentrations are measured in parts per trillion or ng/L [22], and contribute to aroma at these trace concentrations [31,32,33,34]. The detection threshold for IPMP in red wine is 1–2 ng/L [32,34] and 0.3–1.6 ng/L in white wine [34], with similar thresholds reported in grape juices [35]. Factors influencing the IPMP detection threshold in wine—and therefore, how LBT will be experienced—include wine style, mode of evaluation (ortho- vs. retro-nasal assessment), and familiarity with the odorant [34]. For instance, the researchers reported a significantly lower detection threshold for IPMP in a neutral-tasting Chardonnay compared with both a Gewürztraminer and a red blend. Additionally, a trend of greater sensitivity to LBT (i.e., lower detection thresholds) with increasingly familiarity with LBT was observed. The authors suggest that learning of the characteristic sensory cues associated with IPMP occurs in individuals with greater experience of LBT. A corollary, they speculate, is that as awareness of LBT increases in the marketplace, individual thresholds and perhaps acceptance of the taint may decrease [34]. Finally, wine style, mode of evaluation, and familiarity with the taint aside, consumers vary in their sensitivity to IPMP and, thus, how they will respond to wine affected by Coccinellidae; several-hundred-fold differences in detection thresholds have been noted between some individuals [34].

Cudjoe et al. [13] calculated the concentrations of MPs in individual MALBs as ≈27.5 µg/beetle for IPMP, 3.2 µg/beetle for IBMP, and 2.6 µg/beetle for SBMP. What do these values mean for tolerance levels of Coccinellidae in the vineyard? Pickering et al. [8] calculated that 1530 beetles/ton of white grapes or 1260 beetles per ton of red grapes would be needed before LBT could be detected in the subsequent wines. That translates to 1.3–1.5 beetles/kg of grapes; however the authors note that these limits could vary based on grape variety and the processing methods used, and thus recommend a more conservative limit of 200–400 beetles/ton of grapes. Galvan et al. [36], using a different approach, derived similar values, estimating the threshold at which 10% of the population can detect LBT as 1.9 beetles/kg grapes (equivalent to 1900 beetles/t), or 0.27 beetle/grape cluster for Frontenac grapes. Ross et al. [37] similarly estimated the threshold to be 1.8 MALB/kg (1800 beetles/t) in Concord grapes.

The density of MALBs in vineyards at or close to harvest certainly exceeds these “threshold” limits in some years, and this occurs on a periodic basis in some regions, possibly reflecting variations in the population of their preferred prey species. Vineyards in North America located near soybean or grain crops appear to be especially vulnerable. Likely, the harvest of these crops removes principal prey species—such as the aphid Hemiptera: Aphididae—and the beetles migrate to adjacent grapevines in search of further food sources and/or to make use of the vines as potential overwintering sites [3]. The position of “zero tolerance” for ladybugs advocated by some wineries and wine agencies is not supported by the scientific evidence. Coccinellidae do not taint the grapes directly; they must be physically incorporated into postharvest operations. They have long been part of the fauna found in grape vineyards, and only become a threat to wine quality in years when densities are excessive. As detailed above, acceptable levels for these beetles have been determined, and can be used to inform what, if any, interventions are needed by winegrowers.

### 1.3. Coccinellidae Species Responsible

Much of the focus to this point has been on MALBs; are all Coccinellidae equal with respect to their capacity to cause LBT in juice and wine? Cudjoe et al. [13] investigated the relative MP composition of three Coccinellidae species common to North America: *Coccinella septempunctata* (seven-spot), *H. axyridis* (MALB) and *Hippodamia convergens.* IPMP was reported in all three species, with the highest concentration (0.81 µg/mg) in *H. convergens* and the lowest in *C. septempunctata* (0.008 µg/mg), while SBMP and IBMP were above the instrumental limits of detection only in MALBs and *H. convergens*. Kögel et al. [14] also reported on the presence of MPs in *C. septempunctata* and *H. axyridis*, with IPMP found to be the most abundant in both species, followed by SBMP (in *H. axyridis*) and IBMP (in *C. septempunctata*).

Botezatu et al. [20] investigated the relative effects of contamination with seven-spot ladybugs and MALBs on wine quality. They added 0 or 10 beetles of each Coccinellidae species/kg of Vidal and Cabernet Sauvignon grapes at crush, and compared MP concentrations and sensory profiles in the finished wines. The addition of beetles led to similar effects on the sensory profiles of the wines, consistent with ladybug taint, regardless of the species. IPMP concentrations were not significantly different between species for Vidal wines, while in Cabernet sauvignon IPMP was significantly higher in wines made with the addition of seven-spot ladybugs. The difference between this result and that of Cudjoe et al. [13] may be attributed to methodological variations (live beetles in the Botezatu study versus frozen and thawed beetles in the Cudjoe study) and/or the different analytical techniques employed. Kögel et al. [16] also reported increases in IPMP concentrations in wines processed with either MALBs or seven-spot ladybugs.

Given that MALBs and seven-spot ladybugs are probably the most common Coccinellidae species in the majority of the world’s wine regions, these results should underline the importance of monitoring for the presence of ladybeetles in vineyards at harvest. Both species show similar capacity to cause LBT in resulting wines and, thus, do not need to be differentiated in the vineyard when deciding on intervention thresholds, although it is unlikely that seven-spot ladybugs reach the densities required to affect wine quality as often as do MALBs.

## 2. Preharvest Prevention in the Vineyard

Coccinellidae beetles tend to appear in vineyards in the fall, after undertaking dispersal flights from their original feeding habitats both in response to temperature changes and as a result of the crops that initially hosted them being harvested [38]. Once in the vineyard, they do not damage healthy fruit, but they do feed on previously damaged grapes [39]. This underlines the importance of good vineyard management practices in order to prevent or reduce damaged fruit, such as bird-displacement measures, good canopy management, and the proper use of antifungal products. Since aphids are the main food source for MALBs, good weed management late in the season should be observed in order to reduce weed populations that can host aphids attractive to MALBs.

One key element of an integrated pest management strategy against *H. axyridis* in vineyards is effective surveying for beetle densities before harvest. Galvan et al. [36] assessed the usefulness of various sampling plans, and found binomial sampling to be a more accurate method to determine beetle densities than enumerative plans.

**Figure 3:**
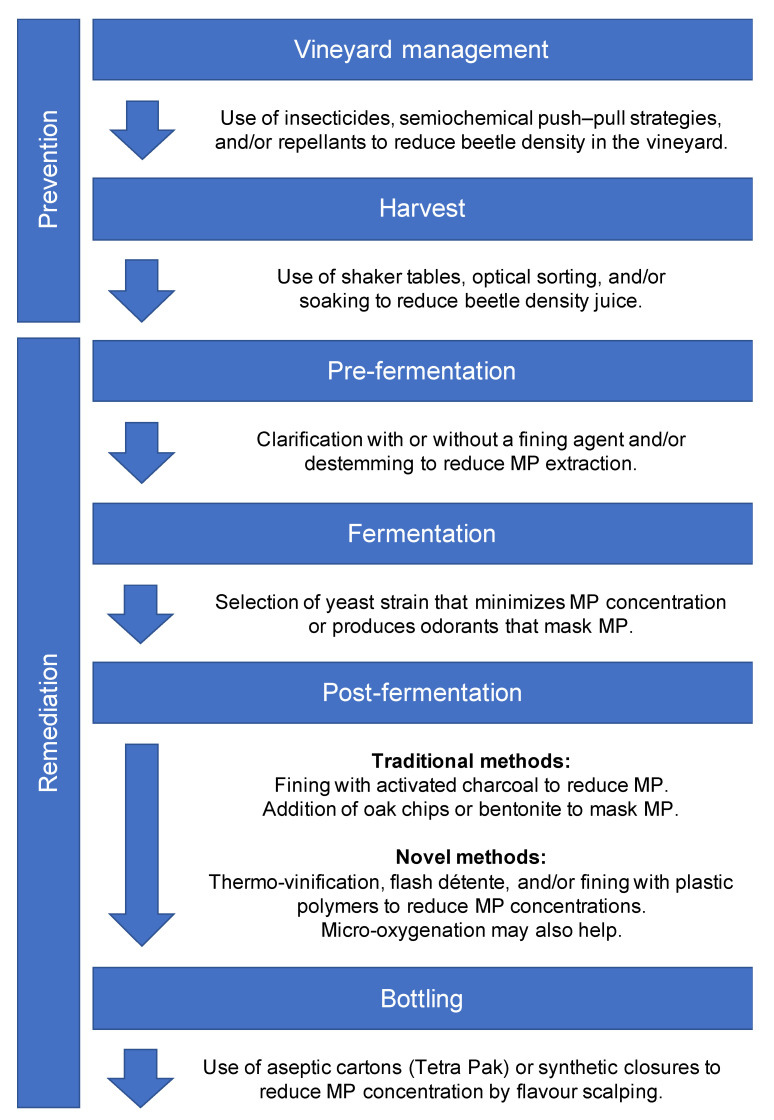
Summary of methods used to prevent or remediate ladybug taint during wine production.

In addition to good vineyard management practices, other methods can be applied to reduce Coccinellidae populations, when detected, to below the tolerance levels outlined in Section 1.2. These fall into three general strategies: insecticides, which kill the beetles; semiochemical push–pull approaches, which combine both repellants and attractants to affect the spatial distribution of beetles; and repellent sprays that drive the beetles away from the vineyard (Figure 3).

### 2.1. Insecticides

Application of insecticides is the most common intervention used in North America to control Coccinellidae in vineyards. In Canada, Malathion 85 E (malathion) and Ripcord™ 400 EC (cypermethrin) can be used with a preharvest interval of seven and three days, respectively. Ripcord™ 400 EC has an extended repellency effect, and both products have good knockdown success [40]. In the United States, accepted insecticides include Venom^®^ 70SG (dinotefuran), Clutch^®^ 50WDG (clothianidin), and Mustang^®^ Max EC (permethrin), with preharvest intervals of 0–1 days [40]. As noted in Section 1, MALBs can still elicit LBT in subsequent wines up to three days after they have been killed [7]; therefore, care should be taken during harvesting operations to minimize their incorporation with the fruit.

An issue with insecticides—particularly those with longer preharvest intervals—is that re-infestation can occur rapidly, since ladybeetles are very mobile and their densities can vary greatly from day to day [41]. Additionally, improper use of these products can increase the potential for elevated or unsafe levels of pesticide residues on grapes, or in juice and wine [42]. These potential shortcomings point to the need for alternative or complementary approaches for controlling MALBs in vineyards.

### 2.2. Semiochemical-Based Push–Pull Strategies

Semiochemical-based push–pull strategies use a combination of highly repellant and highly attractive stimuli in order to control the distribution and abundance of beetles in the vineyard. Ideally, the beetles are “pushed” away from the vineyard by the presence of the repellent compounds, while at the same time are attracted towards other areas–such as trapping zones or protected areas—by the “pull” compounds. Leroy et al. [43] evaluated semiochemicals from aphids (Z,E-nepetalactone, [E]-β-farnesene, α-pinene, and β-pinene), coccinellids ([-]-β-caryophyllene), and the nettle *Urtica dioica L*. as potential attractants for MALB—first in a wind tunnel, and later in a potato field. They reported Z,E-nepetalactone as the most efficient attractant in the wind tunnel experiments, and that MALBs were also responsive to it in the potato field. This latter result suggests that Z,E-nepetalactone may be effective under ecologically valid conditions as an efficient approach for controlling MALBs.

Following the observation that MALB beetles prefer feeding on damaged and overripe grapes [5], Glemser et al. [40] tested grape-derived, nontoxic compounds at two concentrations (high/low) for their potential efficacy as components of a push–pull strategy. They also included MPs in their study, as IBMP, IPMP, and SBMP had previously been identified as attractants to seven-spot beetles [44]. Results showed that MALBs were attracted to acetic acid (both concentrations), acetaldehyde (low concentration), acetic acid plus ethanol, acetic acid plus isobutanol, and IPMP (low concentration, 0.1 ng/L). As part of the same study, but reported elsewhere [42], the beetles were repelled by both ethyl acetate and a mixture of acetic acid plus acetaldehyde. Taken overall, these results indicate the potential for specific compounds and mixtures associated with grape spoilage/fermentation processes to form part of a semiochemical-based push–pull strategy for MALBs, although considerable work remains to operationalize these initial findings.

### 2.3. Repellent Sprays

Glemser et al. [40] looked at the potential of sulfur dioxide (SO_2_) in the form of potassium metabisulfite (KMS) as a repellent compound against MALBs in vineyards. SO_2_ is widely used in the winemaking industry as an antimicrobial agent, an antioxidant, and a general preservative for wine quality. Therefore, using SO_2_ as a spray could be a simple and inexpensive solution, and concerns about residual presence on the grapes would be minimal, given its use in winemaking. The authors found that when sprayed in the vineyard, KMS significantly reduced the number of MALB beetles on grape vines (≈30% fewer with a 5 g/L solution, and 50–60% fewer on vines treated with 10 g/L KMS). However, a later study showed no repellant effects in the field for a 10 g/L KMS solution, with the authors suggesting that stronger winds around the application date may have dissipated the SO_2_ from the treated vines [38]. Encouragingly, KMS (5 g/L solution) sprayed in the vineyards at 14, 7, 3, or 1 day before harvest did not affect free SO_2_ concentrations in the resultant grape juice [40]. Additionally, the authors report no visible phytotoxicity effects on vines post KMS treatment, but do caution against potential environmental pollution issues (e.g., acid rain).

A more recent report from Glemser et al. [38] examined a series of products that were either already registered for use on grapes or had been previously reported as repellant to other insects. In lab-based repellency trials, carvacrol (a monoterpenoid found in essential oils), Timorex Gold (tea tree oil), pine oil, and granite dust were effective at reducing the number of MALB beetles by more than 80%, with pine oil being the most effective, even 72 h after application. However, in the field, only Biobenton (bentonite; up to 39% reduction), Buran (garlic powder + KMS), and Solfobenton (KMS and pine oil) were effective.

Other repellent compounds have also been identified, such as terpenoids from catnip oil (*Nepeta cataria* L.), grapefruit [45], camphor, menthol [46], and even the mosquito repellent DEET (N,N-diethyl-3-methylbenzamide) [47]. However, while suitable for spraying on buildings, these products have not been tested or approved for use in vineyards. Additionally, the potential for residual effects on juice and wine flavor need to be considered for any repellency spray and, thus, sensory analyses should be included in future studies before recommendations on their use can be made.

## 3. Postharvest Prevention and Remediation

When grapes infested with Coccinellidae arrive at the winery, or wine is made and subsequently identified as being affected by LBT, what options are available for fixing the problem? A significant body of research exists around this question, in part because solutions to LBT in many cases will also be transferable for improving wine made from grapes of suboptimal ripeness, given that elevated MP levels are the common cause. Both conventional treatments and newer approaches under development are reviewed below and summarized in Figure 3.

### 3.1. Removing Beetles

Perhaps the most intuitive and simple action is to remove beetles from the grapes at or after harvest prior to further processing. Specialized shaker tables have been designed to facilitate this, and are very effective, although they are generally restricted to hand-harvested fruit, and throughput can be limited [48]. Anecdotally, optical sorters have also been reported to be very effective. These systems use high-speed cameras and image-processing software to separate grapes from beetles (and other non-grape material), and can be incorporated into the grape harvester. Alternatively, some wineries report soaking the grapes in water, allowing for the beetles to rise to the surface, where they can be removed, but this can potentially lead to dilution of the sugar concentration and affect wine quality [48]. Several interventions that are part of traditional winemaking practice have been evaluated in order to determine their effectiveness at removing MPs from must/wine or reducing the severity of LBT.

### 3.2. Traditional Approaches to Treating Faulted Wine

In considering interventions for musts/wines affected by Coccinellidae, the winemaker should also be cognizant of minimizing the contribution from the endogenous MP load of the grapes, especially in high-MP varieties such as Cabernet Sauvignon, Cabernet Franc, and Carmenère, or when the grapes have not reached full flavor maturity (Section 1.1). This includes de-stemming, as over half of grape-derived MPs come from the stems [24,49], and consideration of maceration time, with most MP extraction occurring during the first 24 h [50,51].

Clarification of white wine juice is effective in reducing MP levels by up to approximately 50% of their initial levels [52]. The authors tested Chardonnay juice that had been clarified either through the use of bentonite (1 g/L) or natural settling (24 h and 48 h), and found that regardless of the clarification method, wine produced from clarified juice had significantly lower IPMP concentrations compared with wines produced from unclarified juice. They also showed that the longer the settling period, the greater the decrease in IPMP for both naturally clarified and bentonite-clarified juices.

The use of selected yeast strains to decrease MP levels in wines or mediate their sensorial expression has also been investigated. The abstract of the presentation by Treloar and Howell [53] suggests the ability of some yeast strains to reduce IBMP concentrations in red wine. However, limited data were provided, including no information on the analytical method(s) employed, making it difficult to evaluate these claims. Sorptive processes involving yeast cell walls are known to occur with other classes of volatile compounds (e.g., [54]), so this possibility cannot be discounted for MPs. However, the findings of Pickering et al. [55] provide a cautionary note; they used Cabernet Sauvignon juice spiked with 30 ng/L IPMP, and fermented it with various commercial *Saccharomyces* yeast strains (Lalvin BM45, Lalvin EC1118, Lalvin ICV-D21, and Lalvin ICV-D80). The effects were mixed, with some yeasts unexpectedly increasing IPMP levels (BM45, 29% increase), while others had no effects on IPMP concentration. From a sensorial perspective, the D80 strain produced wines with the highest intensity ratings for LBT-related attributes, while D21 had the lowest ratings. Since MP levels were not affected by these two strains, the effects are most likely attributable to matrix composition and the masking effects of other aroma compounds. The authors recommend D21 as a suitable choice for wines with high IPMP levels, regardless of their source [55]. Sala et al. [50] examined the effects of malolactic fermentation after alcoholic fermentation on MPs, and concluded that malolactic fermentation had no effect on MP levels in the wine.

Fining is a common way of dealing with various wine faults and instabilities, so several fining agents were examined by Pickering et al. [56] as possible LBT remediation options. The authors assessed the application of activated charcoal, bentonite, oak chips, deodorized oak chips, white light, and ultraviolet light in both red and white wines produced with the addition of MALB beetles. The relative efficacy of the treatments varied between red and white wines, with red wines generally more responsive, particularly in terms of flavor improvement. In reds, oak chips did not affect IPMP concentrations, but were the most effective addition in reducing the intensity of LBT-related attributes. Additionally, asparagus/bell pepper flavor was significantly lower in red wines treated with bentonite, charcoal, and deodorized oak. In white wine, activated charcoal reduced IPMP concentrations by 34%; however, LBT-related characteristics were unchanged. Oak chips also led to a significant decrease in asparagus flavor in whites, as well as a trend for lower intensities for all LBT attributes. The positive impact of oak chips on both white and red wines was attributed by the authors to perceptual masking of LBT by other aromatic constituents in the wines, since the addition of deodorized chips did not reduce the intensity of the taint-related attributes. Light had no effect on IPMP concentrations nor sensory profiles in either type of wine.

The sorptive capacity of packaging leading to direct removal of volatile compounds (termed flavor scalping) has been previously established in the food literature, as well as exploited commercially—particularly with polymer packaging and nonpolar flavor compounds [57]. Thus, the possibility that the choice of wine packaging and closure type might reduce MP levels in finished wine has also been investigated as a potentially “noninvasive” approach to remediating LBT.

The effects of both closure and packaging on MPs and other odorants in Riesling and Cabernet Franc wines were investigated by Blake et al. [58]. They spiked each wine with 30 ng/L of IBMP, SBMP, and IPMP, and then bottled and stoppered the wines using five cork-type closures—a natural cork, an agglomerate cork, a roll-on tamper-evident (ROTE) screw cap, an extruded synthetic cork and a molded synthetic closure. A portion of the MP-enriched wines was alternatively stored in aseptic cartons (Tetra Pak). All three MPs were affected by the closure or packaging type to some extent. Wines stored in Tetra Pak cartons had the lowest IPMP concentrations, with a 23% and 41% reduction for Riesling and Cabernet Franc, respectively, possibly due to the migration of IPMP to the aluminum surface layer of the carton, with subsequent adsorption on the oxide layer [58]. Concentrations of SBMP were similarly lower than initial levels in Tetra Pak-stored wines (average decrease of 27%). The authors also reported a 10–21% decrease in IPMP concentration after 18 months of bottle-aging in wines closed with synthetic molded closures. IBMP decreased significantly with 18-month aging in all conditions, with the greatest decrease reported with Tetra Pak and synthetic molded closures, and the smallest change with natural cork [58].

Subsequently, the adsorptive capacity of synthetic corks for MPs has been further demonstrated by Pickering et al. [59]. A Chardonnay wine spiked with 40 ng/L each of IBMP, SBMP, and IPMP was soaked for 140 h with either natural corks, agglomerate corks, molded synthetic closures, or extruded synthetic closures at two different closure addition levels (5 and 10 units/L). All closures significantly reduced MP concentrations in the wine, with the greatest efficacy observed with synthetic closures (70–89% reduction). SBMP was most affected by closure treatments. Unfortunately, sensory analysis of the wines was not performed due to volume restrictions; thus, the sensorial impact of these “treatments” on LBT and overall quality remains to be determined. Finally, these trials show that some MPs do decrease simply as a function of aging. However, as other wine odorants also change and new ones are formed during aging/maturation [60], a strategy of just letting the wines age to remove/reduce LBT before releasing them may not be advisable—indeed, the sensory results (after 10 months aging) of Pickering et al. [9] suggest that it is not, although careful barrel aging in some styles should reduce LBT, as supported by some anecdotal reports to author G.J.P.

### 3.3. Novel Interventions

In addition to the traditional methods for processing wine or treating tainted wine reviewed above, several novel approaches or novel applications of existing technologies, have been evaluated with regard to their capacity to remediate LBT or reduce MP concentrations in juice and wine.

#### 3.3.1. Heat

Thermovinification involves the heating of red musts for a short time to 60–80 °C, which can reduce IBMP content in red wine by 29–67% [61]. More recently, Kögel et al. [16] reported a moderate decrease in all MPs examined (i.e., IPMP, IBMP, SBMP, and DMMP) subsequent to thermovinification of Pinot Noir. However, care should be taken, as this technique may lead to undesirable sensory modifications in wines [62].

Thermoflash, known widely in Europe as flash détente or flash release, is a version of thermovinification used to reduce the fermentation time of red wine and improve quality through increased extraction of tannins, color, and aroma compounds [63]. It works by heating the must of crushed grapes to a set temperature (usually around 185 °F/85 °C), then sending the heated grapes to a high-vacuum chamber, where the temperature decreases sharply and causes the water in the skins’ cells to evaporate rapidly, and the water in the berries to turn to steam. The fast expansion of the steam causes the cell walls of the vacuoles in the skins to explode, leading to immediate color and tannin extraction while also releasing aromatic compounds. The evaporated water (6–10% of initial must volume) is then put through a condenser, and remains as a separate byproduct, or is added back to the must later for fermentation [63]. Non-peer-reviewed data indicate that the IBMP content of Cabernet Sauvignon processed with this technology dropped from 19.2 ng/L IBMP pre-treatment to < 1 ng/L after, while the flash water contained 112.4 ng/L IBMP [64]. Reduction of vegetal notes was also reported in Cabernet Sauvignon wine processed through flash détente in France [65], while winemakers in California reported the presence of easily identifiable MP-related aromas in the water condensate [64]. Similar results from Australia showed that pre-flashed Zinfandel berries had an IBMP content of 8.4 ng/kg, the juice contained less than 3.0 ng/L, and the flash water had 27.8 ng/L. Unfortunately, without peer-reviewed data, the validity of these results remains to be determined, although the purported reproducibility of the findings in multiple countries and varieties suggests that it is an approach with considerable potential for reducing MP loads. Sensory impacts on wines thus treated also need to be further elucidated.

#### 3.3.2. Oxygen

Micro-oxygenation (MOX) is the process of intentionally introducing very small, measured doses of oxygen into wines, in order to bring about desirable changes in color, aroma, and mouthfeel. It requires the use of specialized equipment to regulate the amount of oxygen that is administered [66].

Anecdotal reports from wineries suggest that MOX of wines may be effective at reducing MP levels and/or perceptually masking the effects of LBT in wines. Limited peer-reviewed studies add to these observations: Saenz-Navajas et al. [67] investigated the impact of micro-oxygenation on the sensory properties of Merlot wines, while monitoring viable yeasts and SO_2_ levels. While they did not measure MP levels in the wine, they found that MOX led to a reduction in the green vegetable aromas usually associated with them. Similar results were demonstrated with Cencibel wines by Cejudo-Basante et al. [68] and Cejudo-Basante et al. [69]. Contrary to these findings, Oberhostler et al. [70] reported an increase in some vegetative aromas following MOX in a red wine blend.

#### 3.3.3. The Panacea of Plastics?

Ryona et al. [71] reported silicone to be effective at reducing MP levels in grape juice. Their hypothesis was first confirmed on a model juice, and then demonstrated on four grape juices/musts (a Chardonnay white, a Riesling white, a Cabernet Franc rosé, and a Cabernet Franc red). Reductions in MPs ranged from 53% to 93%, with significant decreases observed immediately after the addition of silicone to wine produced without skin contact. As a caveat, decreases in other volatile aroma compounds were also noted, indicating a need for the sensory evaluation of wines treated with silicone.

Botezatu and Pickering [72] further investigated the capacity of plastic polymers to reduce MP levels in wine. In an initial phase, they treated wines with high levels of IPMP, SBMP, and IBMP (20 ng/L each) with 13 different plastic polymers, allowing for the identification of three potential candidates (silicone, ethylene and vinyl acetate, and a polylactic-acid-based biodegradable polymer) based both on their capacity to reduce MP concentrations and their overall influence on the wines’ sensory profiles. The three polymers reduced overall MP concentrations by 18% (ethylene and vinyl acetate), 28% (polylactic acid), and 30% (silicone). The capacity of these selected polymers to reduce MP levels in red wine was then tested as a function of contact time. Significant decreases in all target MPs were observed after 24 h treatment: polylactic acid reduced IPMP and IBMP concentrations by 52% and 36%, respectively, while silicone reduced IPMP and IBMP by 96% and 100%, respectively. Ethylene and vinyl acetate was less effective in lowering MP levels (7% IPMP and 23% IBMP after 24 h).

Botezatu et al. [73] subsequently investigated the capacity of two plastic polymers—one silicone-based, the other polylactic-acid-based—applied with varying surface areas to reduce concentrations of IPMP, IBMP, and SBMP in a Merlot wine. All surface areas tested (50 cm^2^/L, 200 cm^2^/L, and 600 cm^2^/L) showed reductions in all three MPs, and the polylactic acid polymer was the more effective of the two, with reductions in IPMP concentration of up to 75% at a surface area of 600 cm^2^/L [73]. Analysis of non-target volatile aroma compounds indicated minimal changes; however, results from the sensory evaluation of the treated wines were less clear, often not showing the expected reductions in the intensity of LBT-related descriptors. At the same time, the polymers did not contribute negatively to the aroma or flavor profiles of the treated wines.

#### 3.3.4. Just Nuke It

A more unusual approach to remediating LBT has been reported on by Wilson [74] and Wilson et al. [75], with the application of irradiation to tainted wine. In their pilot study, a dose of 100 Gy was reported to decrease the perceived intensity of LBT; however, the authors did not report on how other sensory attributes of the wines were affected by the treatment, especially with respect to the potential oxidative damage to flavor compounds from such a treatment. We are not aware of any further studies that have investigated this approach.

### 3.4. The Challenge of Specificity

A fundamental limitation of most of the interventions to date aimed at remediating LBT is that they lack specificity for the causal compounds—alkyl-methoxypyrazines. That is, while they may lead to a decrease in MP concentrations through both direct and indirect mechanisms, other—typically desirable—aroma and flavor compounds are also removed from the juice/wine or adversely affected. Two approaches that seek to address this challenge are the use of an odorant-binding protein, and imprinted polymers.

Odorant-binding proteins and major urinary proteins (MUPs) are small extracellular proteins belonging to the lipocalin family, and can bind MPs with micromolar affinities [76,77]. Inglis et al. [78] describe the overexpression of piglet odorant-binding protein (pIOBP) and murine major urinary protein (mMUP2) in the yeast *Pichia pastoris*, their subsequent purification and concentration, and the very high affinity of these proteins for MPs in a grape juice matrix. Subsequent reports have focused on mMUP2, and this protein, when applied to Chardonnay juice, binds IBMP and IPMP, and the resulting complexes can be successfully fined out with bentonite [79]. The effectiveness of mMUP2 is excellent: when applied to juice that is subsequently fined with bentonite and filtered with a 10 kDa polyethersulfone membrane, the system removed > 99% of IPMP and IBMP [78]. However, the performance of mMUP2 within the more challenging matrix of wine, where ethanol and phenolic compounds may reduce its efficacy, remains to be fully elucidated. Additionally, further work is required to scale up the juice-based odorant-binding protein system for use on a commercial scale.

A more recent approach involves the use of magnetic molecularly imprinted polymers to remediate wine with elevated IBMP levels. Molecular imprinting involves the creation of a polymer with selective pockets based on a molecular or biomolecular template. Literature shows that the polymerization of monomers in the presence of a molecular target leads to the formation of corresponding binding sites in the resulting polymer [80]. Liang et al. [81] prepared molecularly imprinted polymers using 2-methoxypyrazine as a template, and incorporated iron oxide nanoparticles as magnetic substrates in order to be able to remove the polymers after treatment with magnets. They also used non-imprinted magnetic polymers for comparison, and assessed both polymers in two different Sauvignon Blanc wines, each spiked with 30 ng/L IBMP. After 30 min of contact time they removed the polymers using a permanent magnet. The authors reported that both the non-imprinted and imprinted polymers adsorbed IBMP, with the imprinted polymers having a higher rate of adsorption (up to 45%, compared with up to 38% for the non-imprinted polymer). Furthermore, they showed that the polymers were washable and reusable for up to five cycles [81].

Further research from Liang et al. [82] investigated the addition of a putative imprinted magnetic polymer both pre- and post-fermentation as a corrective treatment for high concentrations of IBMP in Cabernet Sauvignon grape must, and compared its efficacy to both a non-imprinted magnetic polymer and a polylactic-acid-based film added post-fermentation. Sensory analyses of wines showed that treatment with the putative imprinted magnetic polymer was more effective than the polylactic acid film at decreasing “fresh green” aroma nuances, with no negative impact on the overall aroma profiles reported. Post-fermentation addition of a magnetic polymer removed up to 74% of the initial IBMP concentration, compared with 18% for polylactic acid. Adding the magnetic polymers pre-fermentation removed 20–30% less IBMP compared with post-fermentation addition, and also had less effect on other wine volatiles and color parameters [82]. Theoretically, the same general approach could be used for molecularly imprinting polymers that target IPMP and SBMP—the two main MPs involved in LBT—but the literature is currently lacking on that front.

## 4. Conclusions

Ladybug taint can appear in wine when Coccinellidae species—and especially *H. axyridis*—are incorporated into the grape processing stream after harvest. It is primarily caused by 2-*iso*propyl-3-methoxypyrazine, which originates in the hemolymph of the beetles, and for which the human detection threshold in wine is very low (0.3–2 ng/L). Ladybug taint is characterized by “asparagus”, “bell pepper”, “green beans”, “potatoes”, “herbaceous”, “musty”, and “earthy” descriptors, and varietal attributes are diminished. Several options are available in the vineyard for reducing the density of beetles to acceptable limits—approximately 1200–2000 beetles/ton—depending on beetle species and grape variety. Of these options, several insecticidal sprays and potassium metabisulfite are effective, although some sprays are limited by their pre-harvest intervals.

Several methods for the remediation of tainted grape juice and wine have been investigated, including the use of traditional fining agents, with mixed results. Juice clarification prior to fermentation and the use of oak chips in the wine reduce methoxypyrazine loads and mask ladybug taint, respectively, while thermovinification of juice is also effective. Silicone and polylactic acid also show significant potential for reducing methoxypyrazine levels. A challenge with all of these approaches is the relative lack of specificity for methoxypyrazines, meaning that non-target components of the juice/wine—including desirable aroma compounds—can be adversely affected. Two promising treatments with good specificity for methoxypyrazines are at varying stages of development/commercialization: an odorant-binding protein for use in juice, and molecularly imprinted magnetic polymers for use in either juice or wine. Ladybug taint affects wines from many international winemaking regions, and further research on both prevention and treatment is encouraged, given the invasive nature of *H. axyridis* and its widening global distribution.

## Data Availability

There are no data directly connected to this review paper.
